# Editorial: Pathogen-induced immunosenescence: where do vaccines stand?

**DOI:** 10.3389/fragi.2025.1560009

**Published:** 2025-02-06

**Authors:** Mehrnoosh Doroudchi, Hamed Fouladseresht

**Affiliations:** ^1^ Department of Immunology, Shiraz University of Medical Sciences, Shiraz, Iran; ^2^ Department of Immunology, Isfahan University of Medical Sciences, Isfahan, Iran

**Keywords:** immunosenescence, immunobiography, aging, vaccine, virus, intervention

## Introduction

In this Research Topic, we aim to explore the impact of an individual’s immunobiography, along with the effects of vaccination and/or therapeutic interventions, on the acceleration or deceleration of immunosenescence. Immunobiography refers to the cumulative record of an individual’s immunological experiences, shaped by encounters with various types, doses, intensities, and sequences of antigens throughout their lifetime (Franceschi et al., 2017).

In an insightful review, ‘TB and HIV Induced Immunosenescence: Where do vaccines play a role?’, Singh et al. confer about the mechanisms by which both TB and HIV contribute to immunosenescence. These pathogens are linked to the expansion of CD57^+^CD8^+^ T cells, increased inflammation, hypermethylation of senescence-associated genes, and the shrinking of lymphoid organs. The authors further explore the impact of HIV-*M. tuberculosis* co-infection on the accelerated differentiation of T cells into intermediate effector phenotypes, which contributes to early-onset immunosenescence. The article also addresses the controversial role of the BCG vaccine in TB, as well as its potential effects in the context of HIV-*M. tuberculosis* co-infection and its influence on immunosenescence.

The article, ‘The role of TEMRA cell-mediated immune senescence in the development and treatment of HIV disease’ by Guo et al., begins with an overview of the role of hyperantigenemia in accelerating immune dysfunction during chronic viral infections, subsequently addressing the impairments in mitochondrial function and the amplification of oxidative stress caused by HIV and antiretroviral therapy in patients. The authors also explore how the phenotype of highly differentiated immune effector cells (TEMRA) correlates with the upregulation of cell cycle regulators p16 and p21. They highlight that while TEMRA cells are crucial for immune protection, they also contribute to immunological senescence, resist apoptosis, and eventually adopt a suppressive function. This process can be exacerbated by CMV co-infection and is particularly pronounced in antiretroviral therapy non-responders.

In another thorough review, ‘Human immunodeficiency virus and antiretroviral therapy-mediated immune cell metabolic dysregulation in children born to HIV-infected women: potential clinical implications’, Mataramvura et al. examine the existing evidence on immune-metabolic dysregulation within the mitochondria of HIV-exposed uninfected children (HEU) and HIV-exposed infected children (HEI). The authors explore how early exposure to HIV/ART during infancy disrupts oxidative phosphorylation, reduces ATP production, and enhances the generation of reactive oxygen species, all of which have significant implications for immunosenescence.

In a concise report, ‘Markers of Immunosenescence in CMV seropositive Healthy Elderly Adults', Rodriguez and Parra-Lopez investigate the impact of aging on the innate and adaptive immune cells of CMV seropositive, healthy adults. The authors document an increase in CD14^+^CD16^+^ intermediate monocytes and an expansion of CD56-negative NK cells in elderly individuals.

We trust that this Research Topic will stimulate further discussion and draw attention to this often-overlooked area of research.
